# Structural Obstacles in Saving for Retirement by Race/Ethnicity

**DOI:** 10.1093/geroni/igaf122.1438

**Published:** 2025-12-31

**Authors:** Christian Weller, Dania Francis, Michele Tolson

**Affiliations:** University of Massachusetts Boston, Boston, Massachusetts, United States; University of Massachusetts Boston, Boston, Massachusetts, United States; University of Massachusetts Boston, Boston, Massachusetts, United States

## Abstract

Many people of color have a lot less saved for retirement than White workers, a gap which has remained large for decades. We use the most recent available data to document three structural obstacles to racial retirement equality. Many people of color have less access to an employer-based retirement benefit. They also have lower earnings, which translate into lower retirement benefit contributions. And, they face more widespread and costlier chances of economic setbacks throughout their careers. The data show that the racial gaps in these obstacles are smaller among union members than non-union members. This suggests that greater union membership is one possible policy venue to lower racial retirement wealth inequality. We will discuss other potential employer approaches and policy solutions to increase retirement savings among all groups of households, but especially among Black and Latino householders as well as householders of other/multiple races/ethnicities.

